# Short-term effects of the “Together at School” intervention program on children’s socio-emotional skills: a cluster randomized controlled trial

**DOI:** 10.1186/s40359-016-0133-4

**Published:** 2016-05-26

**Authors:** Olli Kiviruusu, Katja Björklund, Hanna-Leena Koskinen, Antti Liski, Jallu Lindblom, Heini Kuoppamäki, Paula Alasuvanto, Tiina Ojala, Hanna Samposalo, Nina Harmes, Elina Hemminki, Raija-Leena Punamäki, Reijo Sund, Päivi Santalahti

**Affiliations:** Department of Health, National Institute for Health and Welfare, PO Box 30, FI-00271 Helsinki, Finland; Department of Education, PO Box 22, FI-33471 Ylöjärvi, Finland; Standards and Methods, Statistics Finland, FI-00022 Helsinki, Finland; School of Social Sciences and Humanities/Psychology, University of Tampere, FI-33014 Tampere, Finland; Department of Health and Social Care Systems, National Institute for Health and Welfare, PO Box 30, FI-00271 Helsinki, Finland; Centre for Research Methods, Department of Social Research, University of Helsinki, PO Box 18, FI-00014 Helsinki, Finland; Department of Child Psychiatry, University of Turku, FI-20014 Turku, Finland

**Keywords:** Children, Socio-emotional skills, Whole school approach, Intervention, RCT, Intervention dosage

## Abstract

**Background:**

Together at School is a universal intervention program designed to promote socio-emotional skills among primary-school children. It is based on a whole school approach, and implemented in school classes by teachers. The aim of the present study is to examine the short-term effects of the intervention program in improving socio-emotional skills and reducing psychological problems among boys and girls. We also examine whether these effects depend on grade level (Grades 1 to 3) and intervention dosage.

**Methods:**

This cluster randomized controlled trial design included 79 Finnish primary schools (40 intervention and 39 control) with 3 704 children. The outcome measures were the Strengths and Difficulties Questionnaire (SDQ) and the Multisource Assessment of Social Competence Scale (MASCS) with teachers as raters. The intervention dosage was indicated by the frequencies six central tools were used by the teachers. The data was collected at baseline and 6 months later. Intervention effects were analyzed using multilevel modeling.

**Results:**

When analyzed across all grades no intervention effect was observed in improving children’s socio-emotional skills or in reducing their psychological problems at 6-month follow-up. Among third (compared to first) graders the intervention decreased psychological problems. Stratified analyses by gender showed that this effect was significant only among boys and that among them the intervention also improved third graders’ cooperation skills. Among girls the intervention effects were not moderated by grade. Implementing the intervention with intended intensity (i.e. a high enough dosage) had a significant positive effect on cooperation skills. When analyzed separately among genders, this effect was significant only in girls.

**Conclusions:**

These first, short-term results of the Together at School intervention program did not show any main effects on children’s socio-emotional skills or psychological problems. This lack of effects may be due to the relatively short follow-up period given the universal, whole school-based approach of the program. The results suggest that the grade level where the intervention is started might be a factor in the program’s effectiveness. Moreover, the results also suggest that for this type of intervention program to be effective, it needs to be delivered with a high enough dosage.

**Trial registration:**

ClinicalTrials.gov identifier: NCT02178332; Date of registration: 03-April-2014.

**Electronic supplementary material:**

The online version of this article (doi:10.1186/s40359-016-0133-4) contains supplementary material, which is available to authorized users.

## Background

Epidemiological research shows that behavioral, emotional and social difficulties often start at early age with 5–15 % of children and 20–25 % of youth suffering from some mental health problem [1–5]. These difficulties have negative effects on children’s’ quality of life in general and increase the risk of various psychological, physical, and socioeconomic problems, as well as substance abuse and delinquency later in life [6, 7]. Despite the availability, growing use of, and advances in treatments for mental health problems services [8, 9], many children suffering from such problems will not seek or receive treatment, or terminate it prematurely, fail to respond to it, or continue to have difficulties despite treatment [8]. Thus, there is a need for alternative intervention approaches that could reach children and adolescents with mental health problems more widely as well as provide a means for the prevention of such problems.

There is growing evidence of the benefits of prevention and promotion aimed toward reducing the risk of mental health problems and increasing psychological well-being at an early stage and age [8, 10]. Current approaches to prevention include universal interventions, which are targeted to whole child populations regardless of their health or risk status [8, 11, 12]. In schools, practically the whole population of children and adolescents can be reached which makes school a natural environment for universal interventions. Furthermore, the school environments provide stability with an existing school curricula, structures, agreed policies, and resources, which are all essential for well planned, systematic and long-term mental health interventions [13–17]. School-based, universal socio-emotional learning (SEL) programs have been shown to have significant positive effects on children’s socio-emotional skills: according to their meta-analysis of 213 studies, Durlak et al. [14] reported a mean effect size of 0.57 (Hedges’ *g*) for socio-emotional skills, while somewhat smaller effects for other outcomes including social behaviors, conduct problems, emotional distress, academic performance, and attitudes.

Although the importance of prevention has been acknowledged within educational and public policies, there is still much to be done concerning governmental structures and a shared commitment among the respective stakeholders [15, 18]. In Finland, the Ministry of Health and Social Affairs recognized the need for a program promoting children’s socio-emotional skills and mental health in schools and, in 2003, initiated the development of a school-based intervention program. This process resulted in the Together at School intervention program, which is a carefully developed program combining effective components from other school-based programs as well as unique elements developed to fit the Finnish school system and primary-school curriculum [19]. The Together at School intervention was developed in close cooperation with school staff and tested in every-day school work across several years. The aim of the program is to promote children’s socio-emotional skills in a whole school context. The intervention program consists of manualized tools and methods, training of the intervention elements, and school visits by the instructors [20]. The intervention is carried out in classrooms by teachers who are seen as the primary agents of the children’s SEL process. In order to support the SEL process of the children in line with the whole school approach, the intervention also aims to provide similar experiences of SEL to school staff with the help of the principal. Teacher-parent collaboration is also supported.

Earlier research suggests that school based interventions, especially those promoting broader developmental domains enhancing socio-emotional skills, should be started early with the youngest children [16]. In line with this, the Together at School program has focused on the first school years, with the first school year, when the child arrives in a new educational environment, being considered especially important for the training of social relations and emotions. In the present Randomized Controlled Trial (RCT) the Together at School intervention was administered also at the second and third grades, in order to examine whether the program is equally efficient when administered at different grades, and for children of different ages (in Finland first graders are seven, third graders 9 years old).

Concerning intervention implementation, the question whether and to what extent the intervention dosage is related to its effectiveness, is important. Accordingly, the present study analyses the amount that the intervention methods and tools are used in real life school work situations. It has been pointed out that there is a gap in research regarding how the implementation variables interact with the intervention program and affect implementation effectiveness and student outcomes [21]. Moreover, dosage effects have been somewhat underreported, even if implementation quality is considered to be important for both intervention success and as one of the possible explanations for the absence of positive intervention results [22, 23]. Available research suggests that intervention dosage is related to intervention effectiveness and that a higher dosage potentially leads to more positive student outcomes [15, 21].

### Aims

The aims of the study were, first, to examine the short-term effects of the Together at School intervention program, a universal, whole school-based program targeted at improving primary-school children’s socio-emotional skills and reducing psychological problems.

Second, the study examined whether the intervention effects vary depending on the grade (Grades 1–3) the children are in when the intervention program is started. In the view of earlier research our hypothesis is that the intervention is likely to be more effective among younger children, i.e. when started already in the beginning of the child’s school path.

Third, we addressed the question regarding how the intervention dosage is related to intervention effectiveness and our hypothesis is that the intervention would be effective more likely when implemented with the intended intensity.

Finally, while the Together at School intervention is intended to be used among both boys and girls, we were also interested to see whether there are any gender differences regarding the aforementioned study questions. We know from previous literature that boys and girls differ significantly in emotional and social skills and psychiatric problems at elementary school years [24–26]. Thus, in addition to presenting results for the total sample as the primary analysis, we also present data separately for boys and girls.

## Methods

### The context of the present study

Finland is an egalitarian country with a rather high standard of living and relatively small socio-economic differences. It is compulsory to attend school in Finland from the age of seven (Grade 1) until the age of 15 (Grade 9). The school system is financed and organized by local municipalities and regulated by the Ministry of Education and Culture, and only a very small minority of Finnish children attend private schools. To examine the effectiveness of the Together at School intervention program, a cluster RCT was organized. The RCT was conducted in the whole of Finland including schools from different parts of the country. Data was collected at baseline, 6 months^1^ after baseline, and will also be collected 18 months after baseline from the same participants (children and their parents, teachers and the principals). The present study is part of this RCT and focuses on the primary child outcomes (socio-emotional skills and psychological problems) assessed by the teachers at baseline (T0) and 6-month follow-up (T1).

Prior to the RCT, the intervention program went through an excessive development process of several years, during which a group of teachers, principals, and healthcare professionals tested, modified and adopted the intervention methods and tools in close collaboration with three development schools. Moreover, the intervention program was piloted in four schools in four different towns. Analyses of the pilot study indicated that the intervention program was feasible, perceived beneficial and suitable in different school settings [27].

### Ethics approval and funding

The study protocol was approved by the Ethics Committee of the National Institute for Health and Welfare in Helsinki, Finland (27.9.2012) and the trial is registered in the ClinicalTrials.gov registry (NCT02178332). The trial was funded by the Finnish Ministry of Education and Culture, the National Institute for Health and Welfare and the town of Ylöjärvi.

### Recruitment procedure

**Fig. 1:**
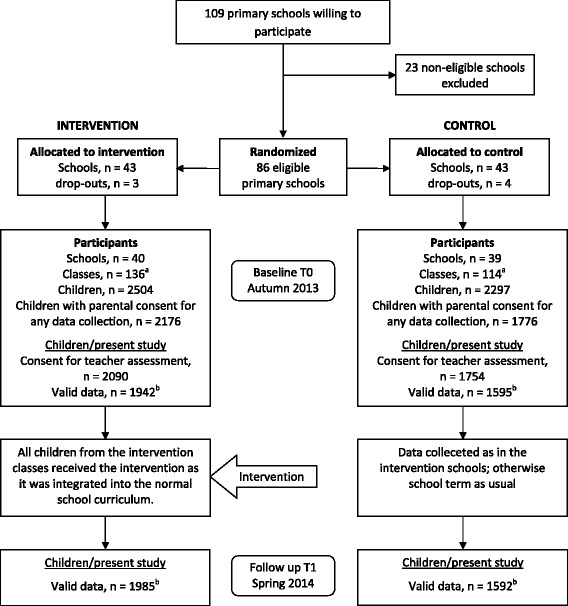
Flow chart of participants. ^a^There were 2 intervention and 6 control group classes where the teacher did not report any data valid for the present study and were thus excluded, leaving 134 intervention and 108 control group classes for the analyses. ^b^All in all there were 2036 (out of 2090) children in the intervention and 1668 (out fo 1754) in the control group, for whom the teacher reported valid data (outcomes) either at baseline or follow up

All Finnish primary schools were invited to participate in the study on the condition that the school had a minimum of two teachers, who agreed to participate for the whole study period of two school years, and who were teaching the first, second or third grades. Of the 109 schools that were willing to participate, 23 were excluded from the study as they were considered non-eligible due to the risk of contamination or excessive training costs. The eligible 86 schools were randomized into either intervention or control groups. After the randomization, seven schools declined their participation due to various reasons (e.g. school economic situation or personnel shortage) resulting in 79 (40 intervention and 39 control) schools in the study. The participant flow is outlined in Fig. 1 and the recruitment process and randomization are reported more in detail in the study protocol [20].

All parents of the participating classes received an information letter regarding the intervention program and aims of the study. The parents were informed about the voluntary nature of the participation in the data collection and a consent form for data collection was included in the information letter. The teachers and principals consented by agreement [20].

The proportion of children with parental consent for data collection was higher in the intervention group (*n* = 2176, 86.9 %) compared to the control group (*n* = 1776, 77.3 %) (Fig. 1). Reasons for participant loss (children without parental consent) were gathered from teachers of ten selected schools with the lowest consent percentages. According to these data, the most common reasons for nonconsent were: difficulties in school/teacher-parent communication, cultural and language challenges, and parental stress especially in large and economically-strained families.

### The Together at School intervention program

The Together at School intervention program employed methods and tools within three areas in order to guarantee the whole school approach. All the methods and tools are designed to be integrated into the normal school curriculum. The first set of methods, carried out in class by the teachers, are designed for the children: Circle time, Do-It-Myself lesson, Do-It-Together lesson, and teacher-child individual discussions. Circle time is a 15 min session consisting of guided greetings (e.g. eye contact, friendly touch), children taking turns in telling others about something important to them, and playing – the aim is to practice children’s communication and emotional skills and enhance classroom climate. The Do-It-Myself lesson is a 10–40 min weekly lesson aimed at practicing children’s skills of independent work: concentrating, focusing on one’s own task and problem solving. In the Do-It-Together lesson children work in small groups to practice cooperation skills. At the beginning of the lesson, children are given a vision of successful teamwork. When needed, help and encouragement are provided by the teacher. Children learn to present their own point of view, listen to others’, take turns, and negotiate. Individual teacher-child discussions (twice a year) where the teacher has a role more as a listener are aimed at creating a good and confidential relationship between the teacher and the child.

The second set of methods and tools, carried out by the principal and the staff, are designed to improve the school work environment (Planning of Collaborative Time, Staff Meeting, Service Station, and Toolkit Session). For example, a Toolkit session (45 min, once or twice a year) held by a staff member offers the teaching staff a possibility to share know-how based on their own interests and expertise, aiming at enhancing occupational know-how among the teaching staff. The third set of methods, the teacher-parent methods, carried out by the teachers are aimed at improving and maintaining a good relationship between the home and school and enhance teacher-parent collaboration. The methods include materials for meeting the parents individually (allowing the parents to express their thoughts freely and give information about their child) and for organizing the Parents’ Evening (aimed to activate teacher-parent interaction and provide support to the parents and the teacher in their child rearing work). For a more comprehensive set of descriptions of the contents and purposes of the methods and tools, see additional file in the study protocol [20].

The intervention group teachers received program training before starting the implementation of the intervention. Six instructors with a degree in pedagogics (trained teachers) were responsible for the intervention program training. The program training consisted of theory and practice of the intervention methods and tools (e.g. lessons, exercises, group discussions) and school visits by the instructors. As part of the training teachers received a 258-page Together at School manual where all the intervention methods and tools are described in detail. The training extended over 10 months and included four modules which proceeded in four waves [20]. After each training module the teachers started to use the methods and tools in their own classes individually.

The control group teachers and headmasters received two 3-hour lessons given by the psychologists and child psychiatrists of the research group. In November 2013 topics were children’s mental health in general, emotions and development of emotional and behavioral regulation. In March 2014 the topics were teachers’ well-being and professional development and how to establish good relationship and to cope with challenging situations with children and their parents. Lectures were offered in four central locations in Finland and they were videotaped to be available for those control group teachers and headmasters who could not attend the meeting. After the intervention study (the RCT) the control group teachers will receive the Together at School manual.

### Measure of intervention dosage

**Fig. 2:**
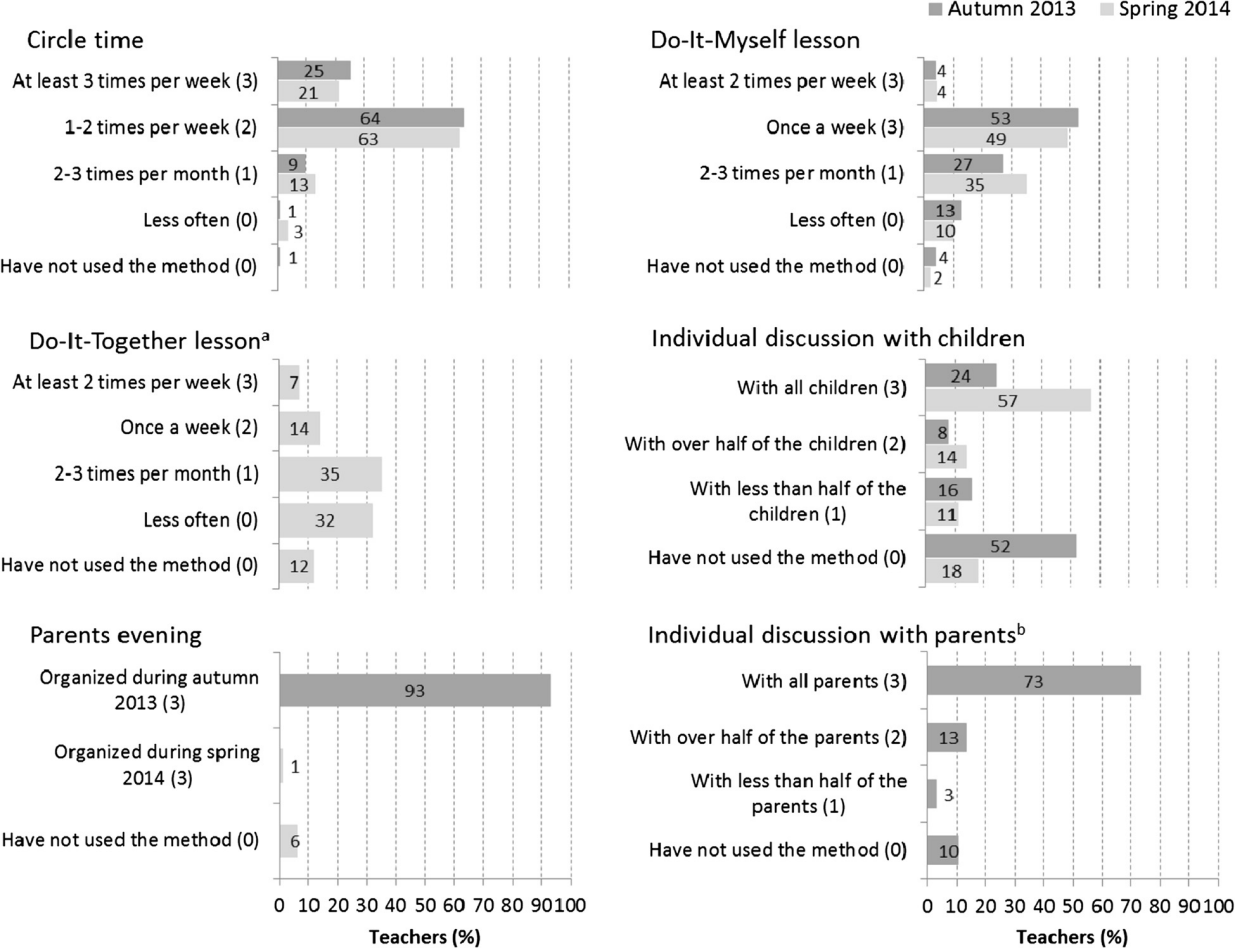
Intervention methods and tools and the frequencies they were used by the teachers during the school terms. For each method and frequency the rating that was used in the calculation of the intervention dosage is given in the parenthesis. ^a^Only in the spring term 2014. ^b^Only in the autumn term 2013

Teachers completed detailed intervention protocols in order to keep a log of the tools and methods they had carried out in their classes [20]. The protocols were used to monitor the implementation process and measure the implementation fidelity, and based on these protocols intervention dosages were calculated. There were four classroom and two teacher-parent methods and tools, six in total, five of which were used in the autumn term 2013 and five in the spring term 2014 (see Fig. 2). The school environment/school staff methods were not included in the measure of dosage in the present study. To calculate the dosage, the intervention tools and methods were all rated first on a scale from 0 to 3 depending on how frequently the teachers had used them in their class during the term so that the maximum value (3) was given when a method was used with the frequency/extent that was specified in the intervention protocol (codes/ratings for the methods are given in Fig. 2). The maximum score for the dosage was 15 (5 x 3) for each term. If dosage was not available for one term due to a missing protocol (19 classes), the dosage of the other term was used as a replacement; two classes with no available protocols were coded to the sample mean dosage value. For the analyses, a mean score of the two terms was calculated and this mean dosage score was then divided into two groups reflecting whether or not the intervention was delivered with the intended intensity (as indicated by the protocol). The dosage groups were named as “intervention below the intended intensity” (0–12.0 points; 78 %) and “intervention as intended” (12.1–15 points; 22 %).

### Measures of outcome

Children’s socio-emotional skills and psychological problems were measured using electronic questionnaires filled in by the teachers at T0 and T1. The Strengths and Difficulties Questionnaire (SDQ) and the Multisource Assessment of Social Competence Scale (MASCS) were used as the primary outcome measures. The SDQ is widely used and has good psychometric properties [28–30]. Also the Finnish version of the SDQ has been shown to have good psychometric characteristics [31–33]. The MASCS measures social competence and it has been designed to fit the Finnish elementary school context [34]. It is partly based on the School Social Behavior Scale (SSBS) [35] and has been validated in Finland [34]. The MASCS includes four subscales (impulsivity, disruptiveness, cooperation, and empathy) of which the two prosocial subscales, cooperation (range 5–20) and empathy (range 3–12), along with the prosocial behavior subscale (range 0–10) of the SDQ, were used to measure children’s socio-emotional skills in the present study. Children’s psychological problems were measured with the SDQ subscales for conduct disorder, hyperactivity, peer relations, and emotional problems, which together formed the SDQ psychological problems measure (SDQ total; range 0–40) [28–30] used in the analyses.

### Statistical methods

All analyses were made first for the total sample, and then separately for boys and girls. Intervention and control group differences in demographic characteristics at T0 were analyzed using chi-square test. Due to the clustered nature of the data the analyses of change between T0 and T1 in the outcome measures (i.e. the intervention effectiveness) were conducted using multilevel modeling with MLwiN Version 2.32 [36]. In clustered data, observations are non-independent, which means that, for example, the responses of the children attending one school class (sharing the same classroom, classmates and the teacher etc.) are more likely to be similar compared with children from a different class. The non-independence within classes might be even more pronounced in the present study, as we used outcome data of the children reported by the (within-class shared) teacher. If this non-independence is not taken into account in the modelling, then there is a possibility of inaccurate standard errors [37].

In the multilevel models, variance was estimated for each dependent variable at four levels: time, children, classes and schools. Also intraclass correlations (ICC), i.e. the proportions of variance each level explains of the total variance, were calculated as indicators of variation among children, classes and schools. While the ICC values at the child level were higher than the class level, they (and corresponding variances) were significant also at the class level indicating that children who share the same classroom were more alike compared to children from other classes. At the school level, the ICC values were low for each dependent variable and the variances were non-significant. Due to this, the school level was excluded from the successive analyses. Thus, a three-level model was fitted to represent change over time and differences between children and classes.

Multilevel models for change over time in socio-emotional skills and psychological problems were made separately for each of the four outcome variable: cooperation (MASCS), empathy (MASCS), prosocial behavior (SDQ) and psychological problems (SDQ). The distributions of the SDQ prosocial behavior and psychological problems scales were skewed, but as the residuals were quite normally distributed no transformation was made to keep the interpretation of the results as clear as possible. The intervention (intervention vs. control), time (T1 vs. T0) and grade (2nd, 3rd vs. 1st) were entered as independent variables. The intervention effect was presented with the Intervention x T1 interaction term (the interaction between group status and time), which can be interpreted as the difference between intervention and control group average change in the outcome measure from time T0 to T1. To examine whether intervention effects were different depending on the grade, the second-order interaction terms Intervention x grade x T1 were introduced to the model. The last set of analyses assessed whether the intervention effects varied depending on the intervention dosage (below/with intended intensity vs. control) using the resulting two interaction terms between intervention dosage and time (intervention below intended intensity/as intended x T1).

### Sample characteristics

As a whole, 242 classes participated in the trial from 79 primary schools (40 intervention and 39 control). The present study sample (*n* = 3704) consisted of all those children who were rated by the teacher either at T0 or T1 on any of the four outcome measures and had parental consent for the teacher assessments. The mean age of the children was 8.1 years (SD = 0.85). As shown in Table 1, there were no major differences in the baseline demographic characteristics between the intervention and control group children or their families, although the proportions of second and third graders were different between the study groups.

**Table 1:** Child demographics by group status at baseline (T0)

Demographic characteristic	Intervention	Control	*p*-value^a^	Total
	n (%)	n (%)		n (%)
N	2036	1668		3704
Gender				
Girls	1020 (50.1)	884 (53.0)	0.08	1904 (51.4)
Boys	1016 (49.9)	784 (47.0)		1800 (48.6)
School grade				
1st	720 (35.4)	607 (36.4)	<0.001	1327 (35.8)
2nd	897 (44.1)	570 (34.2)		1467 (39.6)
3rd	419 (20.6)	491 (29.4)		910 (24.6)
Mother tongue^b^				
Finnish	1496 (95.5)	1190 (96.6)	0.13	2686 (96.0)
Swedish or other	71 (4.5)	42 (3.4)		113 (4.0)
No information, n	469	436		905
Immigrant background^b^	84 (5.4)	86 (7.0)	0.08	170 (6.1)
No information, n	481	442		923
Family type^b^				
Nuclear family	1183 (75.5)	918 (74.8)	0.68	2101 (75.2)
Single parent	175 (11.2)	148 (12.1)		323 (11.6)
Blended family	177 (11.3)	143 (11.6)		320 (11.4)
Other	32 (2.0)	19 (1.5)		51 (1.8)
No information, n	469	440		909
Highest education of the parents^b^				
University of applied sciences or higher	948 (60.7)	727 (59.2)	0.41	1675 (60.1)
Less	613 (39.3)	501 (40.8)		1114 (39.9)
No information, n	475	440		915
Work situation of the parents^b^				
Both parents employed	1070 (68.2)	834 (67.7)	0.78	1904 (68.0)
At least other unemployed	156 (9.9)	131 (10.6)	0.55	287 (10.2)
At least other at home	188 (12.0)	148 (12.0)	0.98	336 (12.0)
At least other studying	112 (7.1)	65 (5.3)	0.04	177 (6.3)
No information, n	467	436		903

^a^Differences between intervention and control group tested using chi-square test

^b^Reported by the parents

## Results

### Descriptive statistics of outcome variables

Descriptive statistics of the outcome variables are given in Table 2. In general, boys had lower scores in socio-emotional skills and higher scores in psychological problems compared to girls. Preliminary comparisons between T0 and T1 scores indicated that there was an overall trend showing a raise in socio-emotional skills and a decrease in psychological problems. Frequencies of cases in the borderline/abnormal category of the psychological problems score (SDQ total) at T0 and T1 are presented in Additional file 1.

**Table 2:** Children’s socio-emotional skills and psychological problems at baseline (T0) and 6 months (T1) by group status, means

		Intervention, mean (sd)		Control, mean (sd)	
		T0	T1	T0	T1
N		1940–1942	1985	1594–1595	1589–1591
Total	MASCS				
	Cooperation	14.79 (3.17)	15.16 (3.20)	14.90 (3.19)	15.18 (3.13)
	Empathy	9.44 (1.90)	9.61 (1.90)	9.52 (1.87)	9.64 (1.80)
	SDQ				
	Prosocial	6.12 (2.42)	6.36 (2.40)	6.33 (2.40)	6.49 (2.41)
	Total/psychological problems	6.31 (5.94)	5.94 (5.66)	5.93 (5.52)	5.69 (5.32)
N		972	987	757–758	743–744
Boys	MASCS				
	Cooperation	14.09 (3.06)	14.35 (3.09)	14.14 (3.06)	14.45 (3.03)
	Empathy	8.99 (1.92)	9.12 (1.94)	9.16 (1.86)	9.25 (1.79)
	SDQ				
	Prosocial	5.36 (2.32)	5.51 (2.39)	5.48 (2.34)	5.59 (2.39)
	Total/psychological problems	7.95 (6.27)	7.62 (6.07)	7.30 (5.83)	7.04 (5.72)
N		968–970	998	837	846–847
Girls	MASCS				
	Cooperation	15.49 (3.12)	15.97 (3.10)	15.60 (3.15)	15.82 (3.08)
	Empathy	9.88 (1.78)	10.09 (1.73)	9.84 (1.83)	9.97 (1.74)
	SDQ				
	Prosocial	6.89 (2.26)	7.20 (2.11)	7.11 (2.18)	7.29 (2.14)
	Total/psychological problems	4.67 (5.08)	4.27 (4.66)	4.68 (4.91)	4.51 (4.63)

Theoretical ranges of the scales: MASCS/Cooperation 5–20; MASCS/Empathy 3–12; SDQ/Prosocial 0–10; SDQ/Psychological problems 0–40

*MASCS* multisource assessment of social competence scale, *SDQ* strengths and difficulties questionnaire

### Intervention effects

Parameter estimates from the multilevel models for intervention effects on children’s socio-emotional skills and psychological problems are presented in Tables 3, 4 and 5. Coefficients for the intervention variable represent the differences between the intervention and control groups at T0. The intervention and control groups did not differ significantly regarding the outcome variables at T0, except for the higher levels of SDQ psychological problems among intervention group boys.

**Table 3:** Intervention effect on school children’s socio-emotional skills and psychological problems, total sample. Regression estimates from multilevel models: intervention effect (Model A, term *Intervention x T1*) and intervention effect moderated by school grade (Model B, terms *Intervention x 2nd/3rd grade x T1*, 1st grade as the reference)

	MASCS				SDQ			
	Cooperation		Empathy		Prosocial		Total/psychological problems	
	Model A	Model B	Model A	Model B	Model A	Model B	Model A	Model B
	Estimate	Estimate	Estimate	Estimate	Estimate	Estimate	Estimate	Estimate
Baseline								
Intercept	14.948****	14.901****	9.512****	9.525****	6.238****	6.172****	6.049****	6.301****
2nd grade	0.014	−0.028	0.070	−0.043	0.137	0.206	−0.337	−0.326
3rd grade	−0.031	0.184	−0.018	0.075	0.246	0.391	0.138	−0.752
Intervention	−0.273	−0.256	−0.162	−0.174	−0.271*	−0.319	0.655*	0.146
Intervention x 2nd grade		0.202		0.131		0.123		0.062
Intervention x 3rd grade		−0.409		−0.133		0.011		1.967**
Change by time								
T1	0.232**	0.358**	0.105*	0.101	0.165*	0.314**	−0.268**	−0.376*
*Intervention x T1*	0.146	0.050	0.060	0.046	0.065	0.113	−0.125	0.144
2nd grade x T1		−0.167		0.082		−0.207		0.046
3rd grade x T1		−0.236		−0.085		−0.266		0.314
*Intervention x 2nd grade x T1*		0.024		−0.030		−0.102		−0.155
*Intervention x 3rd grade x T1*		0.389		0.062		−0.041		−0.867**
Variance components^a^								
Student level								
Intercept	8.521	8.519	2.969	2.969	4.628	4.628	29.013	29.004
T1	4.231	4.231	1.763	1.763	2.435	2.433	8.759	8.758
Class level								
Intercept	1.622	1.615	0.630	0.627	1.146	1.138	4.889	4.733
T1	0.691	0.680	0.245	0.243	0.646	0.633	0.965	0.935

*MASCS* multisource assessment of social competence scale, *SDQ* strengths and difficulties questionnaire

*p < 0.10, **p < 0.05, ***p < 0.01, ****p < 0.001

^a^All variance components were statistically significant (*p* < 0.001)

**Table 4:** Intervention effect on school children’s socio-emotional skills and psychological problems among boys. Regression estimates from multilevel models: intervention effect (Model A, term *Intervention x T1*) and intervention effect moderated by school grade (Model B, terms *Intervention x 2nd/3rd grade x T1*, 1st grade as the reference)

			MASCS				SDQ			
			Cooperation		Empathy		Prosocial		Total/psychological problems	
			Model A	Model B	Model A	Model B	Model A	Model B	Model A	Model B
			Estimate	Estimate	Estimate	Estimate	Estimate	Estimate	Estimate	Estimate
Baseline										
	Intercept		14.125****	14.025****	9.136****	9.073****	5.357****	5.215****	7.433****	7.791****
	2nd grade		0.149	0.178	0.112	0.162	0.178	0.347	−0.668	−0.803
	3rd grade		−0.032	0.284	−0.050	0.106	0.161	0.453	0.535	−0.528
	Intervention		−0.193	−0.053	−0.221*	−0.077	−0.160	−0.024	0.883**	0.155
	Intervention x 2nd grade			0.032		−0.143		−0.085		0.346
	Intervention x 3rd grade			−0.651		−0.368		−0.432		2.452**
Change by time										
	T1		0.261**	0.480**	0.084	0.142	0.139	0.335*	−0.275	−0.360
	*Intervention x T1*		0.032	−0.284	0.042	−0.129	0.029	−0.059	−0.087	0.268
	2nd grade x T1			−0.278		−0.079		−0.346		0.001
	3rd grade x T1			−0.428		−0.109		−0.270		0.280
	*Intervention x 2nd grade x T1*			0.311		0.244		0.129		−0.187
	*Intervention x 3rd grade x T1*			0.841**		0.320		0.190		−1.222**
Variance components^a^										
	Student level									
		Intercept	7.732	7.729	2.994	2.993	4.170	4.169	32.275	32.254
		T1	4.206	4.202	1.859	1.860	2.528	2.526	10.228	10.216
	Class level									
		Intercept	1.678	1.664	0.589	0.584	1.232	1.226	4.893	4.666
		T1	0.827	0.805	0.260	0.252	0.788	0.776	1.166	1.109

*MASCS* multisource assessment of social competence scale, *SDQ* strengths and difficulties questionnaire

*p < 0.10, **p < 0.05, ***p < 0.01, ****p < 0.001

^a^All variance components were statistically significant (*p* < 0.001)

**Table 5:** Intervention effect on school children’s socio-emotional skills and psychological problems among girls. Regression estimates from multilevel models: intervention effect (Model A, term *Intervention x T1*) and intervention effect moderated by school grade (Model B, terms *Intervention x 2nd/3rd grade x T1*, 1st grade as the reference)

			MASCS				SDQ			
			Cooperation		Empathy		Prosocial		Total/psychological problems	
			Model A	Model B	Model A	Model B	Model A	Model B	Model A	Model B
			Estimate	Estimate	Estimate	Estimate	Estimate	Estimate	Estimate	Estimate
Baseline										
Intercept			15.736****	15.681****	9.889****	9.931****	7.024****	6.971****	4.706****	4.973****
2nd grade			−0.288	−0.303	−0.077	−0.255	0.030	0.103	0.228	0.153
3rd grade			−0.106	0.103	−0.037	0.034	0.301	0.393	−0.142	−0.976
Intervention			−0.170	−0.171	−0.004	−0.090	−0.223	−0.332	0.050	−0.460
Intervention x 2nd grade				0.207		0.233		0.138		0.245
Intervention x 3rd grade				−0.344		0.027		0.250		1.634*
Change by time										
	T1		0.209*	0.254	0.118	0.063	0.172*	0.243	−0.261*	−0.369
	*Intervention x T1*		0.251	0.378	0.086	0.200	0.118	0.338	−0.125	0.063
	2nd grade x T1			−0.095		0.203		−0.035		0.001
	3rd grade x T1			−0.042		−0.056		−0.204		0.335
	*Intervention x 2nd grade x T1*			−0.224		−0.224		−0.387		−0.109
	*Intervention x 3rd grade x T1*			−0.109		−0.189		−0.311		−0.538
Variance components^a^										
Student level										
		Intercept	8.015	8.014	2.610	2.612	3.687	3.690	22.086	22.062
		T1	4.065	4.065	1.590	1.589	2.243	2.241	7.281	7.283
Class level										
		Intercept	1.787	1.778	0.649	0.638	1.206	1.187	3.563	3.503
		T1	0.769	0.756	0.315	0.306	0.639	0.613	0.840	0.824

*MASCS* multisource assessment of social competence scale, *SDQ* strengths and difficulties questionnaire

*p < 0.10, **p < 0.05, ***p < 0.01, ****p < 0.001

^a^All variance components were statistically significant (*p* < 0.001)

The first set of models (Models A, Tables 3, 4 and 5) addressed the question of an intervention effect on the outcome variables across all grades by studying the Intervention x T1 interaction terms. The positive values of these interaction terms indicate that the average change from T0 to T1 corresponds to a larger increase in the outcome variable in the intervention group compared to the control group; similarly negative values indicate a relatively larger decrease in the outcome in the intervention group. Thus, for the intervention to be effective the Intervention x T1 interaction terms need to be positive (and significant) on the socio-emotional skills outcomes and negative on the psychological problems outcome. Inspection of these terms in Models A (Tables 3, 4 and 5) indicated no significant intervention effects.

In the second set of models (Models B, Tables 3, 4 and 5), the moderating role of grade on the intervention effect was examined using interaction terms between intervention, grade and time. These models indicated differences in intervention effects on SDQ psychological problems between third and first graders (the reference group) as marked by the significant Intervention x 3rd grade x T1 interaction term (Table 3). Stratified analyses by gender indicated further, that this interaction term was prevalent only among boys and that among them also the corresponding interaction term relating to MASCS cooperation skills between third and first graders was significant (Table 4). To interpret these interaction terms, separate models were specified for each grade level among boys. The results from these models showed that the intervention was effective in reducing psychological problems among third grade boys (regression estimate −0.994, *p* = 0.025), while among first grade boys the effect was close to zero and non-significant (0.294, *p* = 0.364). Regarding cooperation skills, the intervention had a marginally significant positive effect on increasing them among third grade boys (0.528, *p* = 0.078), whereas for the first graders the effect was slightly negative, but again not significantly different from zero (-0.328, *p* = 0.234). There were no other significant Intervention x grade x T1 interaction terms among boys or girls, indicating no other intervention effects moderated by grade. This was also tested between the third and second grades in additional models (not shown).

The last set of analyses examined the moderating role of the intervention dosage on the intervention effects (Table 6). As indicated by the non-significant dosage x T1 interaction terms, intervention implemented below the intended intensity level was not effective, which means that changes in the outcome measures in this group were not significantly different from the changes that took place in the control group. However, among girls the group who received the intervention as intended showed a significant increase in MASCS cooperation skills (interaction term estimate 0.586, *p* = 0.018) and a marginally significant increase in SDQ prosocial behavior (0.404, *p* = 0.053) compared to the control group girls. Similar results were observed for the total sample. Among boys, the intervention effects for the group who received the intervention as intended were in the expected direction, but did not reach the level of statistical significance.

**Table 6:** Intervention effect on school children’s socio-emotional skills and psychological problems moderated by intervention dosage, regression estimates from multilevel models. Intervention effect moderated by levels of intervention dosage (intensity) with control group as the reference: terms *Intervention below/intended intensity x T1*

		MASCS		SDQ	
		Cooperation	Empathy	Prosocial	Total/psychological problems
		Estimate	Estimate	Estimate	Estimate
Total					
Baseline					
	Intercept	14.949****	9.513****	6.238****	6.034****
	2nd grade	0.008	0.069	0.136	−0.288
	3rd grade	−0.030	−0.017	0.247	0.130
	Intervention below intended intensity	−0.255	−0.152	−0.237	0.788**
	Intervention as intended	−0.326	−0.195	−0.388	0.123
Change by time					
	T1	0.232**	0.105*	0.165*	−0.268**
	*Intervention below intended intensity x T1*	0.068	0.031	−0.008	−0.069
	*Intervention as intended x T1*	0.421**	0.162	0.326*	−0.324
Boys					
Baseline					
	Intercept	14.128****	9.135****	5.357****	7.412****
	2nd grade	0.141	0.112	0.178	−0.601
	3rd grade	−0.030	−0.050	0.162	0.525
	Intervention below intended intensity	−0.197	−0.200	−0.137	1.089**
	Intervention as intended	−0.170	−0.297	−0.237	0.060
Change by time					
	T1	0.261**	0.084	0.139	−0.276*
	*Intervention below intended intensity x T1*	−0.019	0.003	−0.032	−0.009
	*Intervention as intended x T1*	0.219	0.182	0.249	−0.372
Girls					
Baseline					
	Intercept	15.736****	9.890****	7.023****	4.700****
	2nd grade	−0.290	−0.080	0.031	0.247
	3rd grade	−0.104	−0.036	0.303	−0.145
	Intervention below intended intensity	−0.132	−0.003	−0.174	0.093
	Intervention as intended	−0.289	−0.006	−0.384	−0.122
Change by time					
	T1	0.209*	0.118	0.172*	−0.261*
	*Intervention below intended intensity x T1*	0.152	0.066	0.033	−0.089
	*Intervention as intended x T1*	0.586**	0.153	0.404*	−0.246

*MASCS* multisource assessment of social competence scale, *SDQ* strengths and difficulties questionnaire

*p < 0.10, **p < 0.05, ***p < 0.01, ****p < 0.001

Note: Variance components at student and class levels (not shown) were all significant (*p* < 0.001)

## Discussion

The Together at School program was designed for primary-school children in order to promote socio-emotional skills and prevent psychological problems in a whole school context. The findings reported here represent the first results concerning the short-term effectiveness of this universal school-based program.

In their meta-analysis of school-based universal SEL intervention programs Durlak et al. [14] reported significant effects of these programs in increasing socio-emotional skills and also in reducing conduct problems and emotional distress, although to a lesser degree. As a whole, we found no similar intervention effects of the Together at School program in improving primary school children’s socio-emotional skills or in reducing their psychological problems 6 months from the baseline. The lack of main effects in our study may be due to the short follow-up period. It is well known that behavioral changes may require a relatively long learning period and/or that they may appear only later on [38]. Similarly, it takes time and energy on the part of the teachers and principals to take in, process and implement a new method in the school curriculum, which might also explain the lack of intervention effects at this point. Indeed, the idea behind this universal whole school intervention program is to produce mental health effects in the longer term by incorporating the program into the teachers’ and school staff’s continuous daily work practices, eventually becoming an integral part of the school curriculum and children’s school environment. Thus, the intervention is likely to need a longer time to display the positive effects it was planned for, and our future task will be to evaluate the program’s effectiveness after a longer time period at the forthcoming 18-month follow-up point.

On the other hand, positive intervention effects have been reported already after relatively short intervention periods, for example in a classroom-based intervention (Incredible Years) for preschool children [39]. However, comparing the studies is difficult, because the settings, targeted children (all vs. selected most problematic), and the age groups, have been quite different. Especially, in a universal intervention, like the Together at School, where the whole group is targeted, and also the targeted behaviors are at a reasonably good level to begin with, any improvements are likely to be smaller and/or require more time to develop, whereas targeting only higher risk children would produce more dramatic intervention effects [16]. Were we to analyze only those children with the lowest levels of socio-emotional skills or highest levels of psychological problems at baseline, we might have been able to observe stronger effects already at this point.

While not showing intervention effects across all grades, our analyses indicated that the Together at School program was effective among third graders in reducing their psychological problems. Stratified analyses showed that this effect was significant among boys only, and that among them the intervention seems to be effective also in improving third graders’ cooperation skills, although regarding this latter effect the results were only suggestive. This result was somewhat unexpected, as previous reviews had recommended starting school interventions, particularly those aiming to develop generic social and emotional skills, early with young children [16]. Since there should not be large qualitative developmental differences between first and third grade boys (i.e. between 7 and 9 year old boys), our result is likely to be related either to the contents of the intervention itself or the school system, or both. The latter might be more relevant here, since in the Finnish school system there is a “leap” between the second and third grades as the curriculum becomes more academically oriented from the third grade onwards. Moreover, more complex socio-emotional skills are required from the children as they move from first to second, and then to the third grade. It might be that for boys these changes are more challenging, thus leaving more room for a SEL intervention to have positive effects among them in the third grade. In line with this, one possible explanation for the absence of similar findings among girls might be their higher levels of socio-emotional skills and lower levels of psychological problems already at baseline.

The question whether and to what extent factors relating to intervention implementation play a role in intervention effectiveness has been recently brought up in the literature [15]. In the present study we addressed this question by analyzing the modifying role of intervention dosage, i.e. the amount of intervention methods and tools used by the teachers in their classes, on the intervention effectiveness. We expected the intervention to be effective more likely when administered with a high enough dosage. In line with this, the results indicated that when the intervention was carried out with the intended intensity, intervention effects were found on cooperation skills. The stratified analyses indicated further, that this effect on cooperation skills was significant only in girls, and that among them the intervention had also a marginally significant effect on prosocial behavior when delivered with the intended intensity. No effects were observed when the intervention was implemented below the intended intensity level. These results are in line with some earlier studies suggesting that a high enough dosage is needed for the intended intervention effects to occur [16, 21]. This may be the case especially in the universal and whole-school approaches, where the quality of school environment is considered to be an integral part of positive child development [16]. An important issue in our future studies will be to develop a finer grained analysis of which methods and tools of the Together at School intervention program are the most relevant for positive intervention effects, and at which level of dosage. While the results of the present study represent only the first short-term findings, they suggest however, at face value, that intervention efforts should be carried out with fidelity and commitment, while there seems to be no point in using these methods only to a low or moderate degree. This finding, especially if it prevails in the longer follow-up analyses, would also be important when making decisions at the policy level–in other words, it would only be worthwhile investing in the program if the required resources for its proper implementation are allocated.

### Strengths and limitations

The strengths of this study are found in the fact that the Together at School intervention program has been carefully developed and tested for several years in a real world school context. Moreover, the program has been adapted specifically for the national school system and culture. We also consider it important that the program is based on the whole school approach and that the methods are integrated as an imminent part of the curriculum, aimed toward instigating profound long-term changes in the practices and ethos of the school.

The strengths of the present study were also found in the large sample size and randomized-controlled study design. The proportion of children with parental consent to participate in the data collection was relatively high (82.3 %). According to teacher reports from ten selected schools with the lowest consent percentages, the reasons for nonconsent related usually to difficulties in school/teacher-parent communication, cultural/language challenges, or parental economic stress. This might be an indication of selective non-response, and as such might have had some influence on the results (possibly lowering the observed effects). The outcome measures that were used have been validated in the Finnish context.

A limitation of the present paper is that we used only the teacher ratings of the children: the fact that the teachers both delivered the intervention and rated the children could have led to some bias, which should be kept in mind when interpreting the results. The decision to use only teacher rating data at this point was made as the parent rating data included more missing information and would have led to a considerable reduction in the number of cases in our analyses.

Contamination poses a possible risk in RCTs diminishing any observable effects of a potentially effective intervention. To avoid this we conducted randomization at the school level. It has also been indicated that contamination is less likely when the intervention method itself is rather complex and/or aims at behavior changes [40]. On the other hand our control group was not a “pure” no-treatment group, but was given lectures on the same themes that the intervention was targeting. In addition, the lecturers reported (from informal discussions with the lecturers) that during the lectures the control group teachers shared actively with each other their experiences of supporting children’s wellbeing and social and emotional skills, indicating that some of them already used some kind of methods comparable to the intervention and also that they were highly motivated in the topics related to supporting child’s socio-emotional development (also evident by their being participants of the study, albeit in the control group). These characteristics of the control condition could have made it less optimal for the intervention effects to be observed.

The follow-up period was short, being in practice between 4 and 6 months. Nevertheless, the results are in line with the prior feasibility study [27] providing further reassurance that the intervention program can be considered as safe in that there were no negative effects in the studied outcomes. As the recent study by Stallard et al. [41] have pointed out, it is important to keep in mind that interventions can also be potentially harmful. That some of our analyses were not specified as primary or planned analyses in the study protocol [20] is a limitation. This holds for both the separate analyses for boys and girls as well as the analyses of the moderating role of dosage on the intervention effectiveness.

In addition to the studied modifiers of the intervention effect (grade level and intervention dosage), also other factors regarding program implementation and teacher behavior may have been contributing to the program effects. For example, those teachers implementing the intervention with the intended intensity might differ in other relevant aspects (e.g. motivational, personality characteristics, etc.) from their colleagues who implemented the intervention below the intended level, and this induces the possibility that the effects in the outcomes are not totally related to the intervention itself. Therefore, more detailed analyses are needed regarding whether the interplay between intervention-related variables and the aforementioned teacher characteristics, as well as the children’s background characteristics, e.g. prior socio-emotional skills and psychological problem levels, has an influence on the intervention program effect. These factors remain as important study questions for further studies.

## Conclusions

The present study reported the short-term results of the Together at School intervention program, a universal school intervention on children’s socio-emotional skills delivered by teachers under real world conditions and integrated to normal classroom education and the school curriculum in a whole school context. No main intervention effects were observed after a 6 month intervention period. Those in the third grade, especially the boys, seemed to benefit from the program, indicating that the grade level where the intervention program is implemented might be a factor in the program’s effectiveness. The results also indicate that for this type of universal intervention program to be effective, it is important that the intervention is delivered with a high enough dosage. These and other modifiers of the potential effectiveness of the Together at School intervention program as well as its long-term effectiveness will be addressed in up-coming follow-up studies.

## Additional files

Additional file 1: Frequencies of psychological problems (SDQ total) by gender, group status and grade at baseline (T0) and 6 months (T1). (DOC 100 kb)

## Abbreviations

ICC: intraclass correlation
MASCS: multisource assessment of social competence scale
RCT: randomized controlled trial
SD: standard deviation
SDQ: strengths and difficulties questionnaire
SEL: socio-emotional learning
SSBS: school social behavior scale

## Footnotes

1 Due to practical and organizational reasons the baseline phase of the study spanned over a period of two months. Consequently the follow-up times varied in practice between 4 and 6 months. For the sake of clarity we refer to this 4–6 month measurement point as “6 months” in the text.
