# Supplementary material for: Short-term effects of the “Together at School” intervention program on children’s socio-emotional skills: a cluster randomized controlled trial
Source: BMC Psychol. 2016 May 26;4:27. doi: 10.1186/s40359-016-0133-4 (PMC4882817; doi:10.1186/s40359-016-0133-4)
Supplement: Additional file 1: — Frequencies of psychological problems (SDQ total) by gender, group status and grade at baseline (T0) and 6 months (T1). (DOC 100 kb) [file 40359_2016_133_MOESM1_ESM.doc]

**Additional file 1: Frequencies of psychological problems (SDQ total) by gender, group status and grade at baseline (T0) and 6 months (T1), %.**

|  |  | Boys | | | | |  | Girls | | | | |
| --- | --- | --- | --- | --- | --- | --- | --- | --- | --- | --- | --- | --- |
|  |  | Intervention, % (n) | |  | Control, % (n) | |  | Intervention, % (n) | |  | Control, % (n) | |
|  | SDQ total^a^ | T0 | T1 |  | T0 | T1 |  | T0 | T1 |  | T0 | T1 |
|  |  |  |  |  |  |  |  |  |  |  |  |  |
| All | Normal | 74.9 (706) | 76.0 (717) |  | 79.5 (571) | 81.1 (582) |  | 90.4 (856) | 92.7 (878) |  | 90.9 (726) | 91.9 (734) |
|  | Borderline/  abnormal | 25.1 (237) | 24.0 (226) |  | 20.5 (147) | 18.9 (136) |  | 9.6 (91) | 7.3 (69) | ** | 9.1 (73) | 8.1 (65) |
|  | Total | 100.0 (943) | 100.0 (943) |  | 100.0 (718) | 100.0 (718) |  | 100.0 (947) | 100.0 (947) |  | 100.0 (799) | 100.0 (799) |
|  |  |  |  |  |  |  |  |  |  |  |  |  |
| 1st grade | Normal | 76.9 (276) | 77.7 (279) |  | 78.6 (213) | 79.7 (216) |  | 91.4 (297) | 92.9 (302) |  | 90.5 (256) | 91.9 (260) |
|  | Borderline/  abnormal | 23.1 (83) | 22.3 (80) |  | 21.4 (58) | 20.3 (55) |  | 8.6 (28) | 7.1 (23) |  | 9.5 (27) | 8.1 (23) |
|  | Total | 100.0 (359) | 100.0 (359) |  | 100.0 (271) | 100.0 (271) |  | 100.0 (325) | 100.0 (325) |  | 100.0 (283) | 100.0 (283) |
|  |  |  |  |  |  |  |  |  |  |  |  |  |
| 2nd grade | Normal | 76.5 (299) | 76.0 (297) |  | 80.4 (180) | 84.4 (189) |  | 91.2 (384) | 92.4 (389) |  | 89.6 (251) | 90.7 (254) |
|  | Borderline/  abnormal | 23.5 (92) | 24.0 (94) |  | 19.6 (44) | 15.6 (35) | + | 8.8 (37) | 7.6 (32) |  | 10.4 (29) | 9.3 (26) |
|  | Total | 100.0 (391) | 100.0 (391) |  | 100.0 (224) | 100.0 (224) |  | 100.0 (421) | 100.0 (421) |  | 100.0 (280) | 100.0 (280) |
|  |  |  |  |  |  |  |  |  |  |  |  |  |
| 3rd grade | Normal | 67.9 (131) | 73.1 (141) |  | 79.8 (178) | 79.4 (177) |  | 87.1 (175) | 93.0 (187) |  | 92.8 (219) | 93.2 (220) |
|  | Borderline/  abnormal | 32.1 (62) | 26.9 (52) | + | 20.2 (45) | 20.6 (46) |  | 12.9 (26) | 7.0 (14) | * | 7.2 (17) | 6.8 (16) |
|  | Total | 100.0 (193) | 100.0 (193) |  | 100.0 (223) | 100. (223) |  | 100.0 (201) | 100.0 (201) |  | 100.0 (236) | 100.0 (236) |
|  |  |  |  |  |  |  |  |  |  |  |  |  |

^a^ SDQ total categories: scores 0-10 = “Normal”; scores 11 or higher = “Borderline/abnormal”

^+^p < 0.10, *p < 0.05, **p < 0.01; changes between T0 and T1 tested with McNemar test.

SDQ = Strengths and Difficulties Questionnaire
